# ATF4-dependent heme-oxygenase-1 attenuates diabetic nephropathy by inducing autophagy and inhibiting apoptosis in podocyte

**DOI:** 10.1080/0886022X.2021.1936040

**Published:** 2021-06-22

**Authors:** Shizhu Yuan, Xudong Liang, Wenfang He, Mingzhu Liang, Juan Jin, Qiang He

**Affiliations:** a The Second Clinical Medical College, Zhejiang Chinese Medical University, Hangzhou,P.R. China; b Department of Nephrology, The First People’s Hospital of Hangzhou Lin'an District, Affiliated Lin'an People's Hospital, Hangzhou Medical College, Hangzhou, China; c Department of Nephrology, People’s Hospital of Beilun District, Ningbo, P.R. China; d Department of Nephrology, People’s Hospital of Hangzhou Medical College, Zhejiang Provincial People’s Hospital, Zhejiang, P.R. China

**Keywords:** Podocyte, autophagy, HO-1, ATF4, diabetic nephropathy

## Abstract

**Aim:**

Podocyte injury plays an important role in diabetic nephropathy (DN), yet the underlying molecular mechanisms of podocyte injury in DN is not clear. Here, we investigated the role of activating transcription factor 4 (ATF4) and HO-1 in DN-induced podocyte injury.

**Methods:**

Protein expression was measured by western blotting (WB) and immunofluorescence. Cellular apoptosis was quantified by flow cytometry. ATF4 siRNA knockdown and HO-1 overexpression in podocyte were employed to evaluate the role of ER stress in DN-induced apoptosis and autophagy response. Urinary protein levels, nephrin expression, serum creatinine and BUN were evaluated and glomerulosclerosis was quantified by Periodic Acid-Schiff staining.

**Results:**

Expression of ATF4 was increased in podocytes exposed to serum from DN mice. ATF4 knockdown enhanced DN-induced podocyte apoptosis. HO-1 overexpression reduced the decline of DN-induced podocyte autophagy and inhibited apoptosis and the beneficial effects of HO-1 overexpression in DN were blocked by ATF4 knockdown. The diabetic mice were significantly ameliorated by HO-1 agonist hemin treatment.

**Conclusions:**

ATF4 induces autophagy by enhancing the expression of HO-1, and inhibits podocyte apoptosis in DN. Treatment with the HO-1 agonist reduced proteinuria, apoptosis, and enhanced autophagy response, and thus improved renal function in DN mice.

## Introduction

Diabetic nephropathy (DN) is a serious complication associated with diabetes. The primary indication of DN is proteinuria. DN is a risk factor for kidney disease progression and if left untreated, it can lead to end-stage renal disease (ESRD) [1,2]. Podocytes or glomerular epithelial cells are terminally specialized cells that play a critical role in forming the glomerular filtration barrier. Structural changes or injuries in podocytes are associated with renal injury resulting in proteinuria and severe renal insufficiency [3–6], and contributes to DN [7].

Autophagy is a highly conserved process that utilizes the lysosomal pathway to degrade and recover cellular proteins and to remove and recycle damaged organelles. When autophagy is activated, cell homeostasis is maintained [4,8–11]. In DN, pathogenic molecules present in the serum is shown to inhibit autophagy and induce apoptosis in podocytes [12]. Previous studies have shown that podocyte dysfunction may lead to proteinuria *via* activation of epithelial-to-mesenchymal transition (EMT). By blocking EMT, podocyte apoptosis can be reversed [13–15].

Endoplasmic reticulum (ER) stress is triggered by the accumulation of unfolded proteins in the cells, and thus activates the unfolded protein response (UPR) which is associated with apoptosis in podocytes. Activation of ER stress plays an important role in the development of DN [16]. Accumulating evidence has shown that ER stress also induces autophagy as a mechanism to protect the cells from entering apoptosis. UPR may alleviate misfolded protein mediated ER stress by initiating autophagy [17]. In UPR, the PERK-eIF2α-ATF4 pathway plays critical role in regulating ER stress and restores protein homeostasis PERK (Protein kinase RNA-like endoplasmic reticulum kinase) is a pivotal transducer of ER stress by phosphorylating eukaryotic initiation factor 2 α-subunit (eIF2α), resulting in promoting pro-adaptive signaling pathways by decreasing global mRNA transcription and activation of ATF4 transcription factor [18–20]. Activation of PERK-eIF2α-ATF4 signaling pathway in ER stress has been found to be associated with autophagy induction and maintenance of intracellular homeostasis. However, growing evidence suggest that impairment in autophagy during prolonged ER stress and failure of pro-adaptive responses may lead to programmed cell death or apoptosis in DN [21,22]

Heme oxygenase-1 (HO-1) is an essential enzyme in heme catabolism that has been shown to protect cells by inhibiting oxidative stress and apoptosis [23]. Previous studies have shown that ATF4 regulates HO-1 expression [24,25], however, role of ATF4/HO-1 pathway in regulating autophagy and apoptosis in DN is not defined. In the present study, we demonstrated that ATF4 regulates autophagy in DN podocytes by promoting HO-1 expression. siRNA mediated knockdown of ATF4 led to autophagy impairment and increased apoptosis in renal podocytes, which were partially rescued by mTORC1 inhibitor rapamycin and HO-1 overexpression in podocytes *in vitro*. Additionally, HO-1 agonist hemin treated DN mice showed marked reduction in albuminuria and renal damage accompanied by autophagy induction and reduced apoptosis in the kidney. Our present study utilizing *in vitro* and *in vivo* approaches shed light on the biology of ATF4/HO-1 pathway in regulating autophagy and apoptosis in renal podocytes in DN and suggests beneficial therapeutic effect of HO-1 agonist hemin in treating diabetic nephropathy.

## Materials and methods

### In vitro stuies

Mouse podocyte cells, MPC5, were purchased from the Institute of Basic Medicine, Chinese Academy of Medical Sciences (Beijing, China) (3111C0001CCC000230). Podocytes were cultured in RPMI 1640 medium (Hyclone, SH30809.01B) supplemented with 10% fetal bovine serum (FBS, 11 nM D-glucose; Gibco, 16000-044) and subcultured at a 1:4 split. For propagation, podocytes were cultured at 33 °C and supplemented with 20 U/mL recombinant murine interferon-γ (IFN-γ, sigma, I4777). Podocytes were then cultured with 1.5 mL/25 cm^2^ collagenase IV (Gibco, 17104-019) for one hour, and washed with 3 mL/25 cm^2^ phosphate-buffered saline (PBS, Solaibao, P1020) until the podocytes retracted and began to detach from the bottom of culture flask. After cultured at 37 °C for 10 days, podocytes were subcultured at a 1:2 split in RPMI 1640 medium containing 10% FBS and 5 mM D-glucose for 24 h and then seeded onto 6-well plates, adjusting the concentration to 1 × 10^6^ cells/ml. Every well contains 0.5 mL podocytes suspension, and 1.5 mL RPMI-1640 medium supplemented with 10% fetal bovine serum. Podocytes were then cultured at 37 °C and 5% CO_2_ atmosphere for 24 h. Then MCP5 podocytes were treated either with 10% serum from control C57BL/KsJ dm/m mice or with 10% serum from C57BL/KsJ dm/dm DN mice for 24 h.

### In vivo studies

#### Group sizes and treatment

Animal experiments conformed to institutional standards. Eight-week-old male db/db mice exhibiting spontaneous DN (*n* = 6) and control C57BL/KsJ-db/db mice (*n* = 12) were raised to 13 weeks and divided into three groups: blank control group (*n* = 6); model-db/db group (*n* = 6); and treatment-db/db group (*n* = 6). Treatments started 24 h after urine was collected at 13 weeks of age. The db/db treatment group was injected intraperitoneally with hemin (25 mg/kg body weight each time, three times a week), while the db/db model group and blank control group were injected intraperitoneally with an equivalent volume of vehicle.

#### Construction of HO-1 overexpression vectors

Purified HO-1 gene fragments were digested with BamHI-EcoRI and ligated into pcDNA3.1(+) vector, which resulted in a recombinant plasmid called PCDNA3.1(+)-HO-1. Competent *E. coli* DH5α cells were transformed with the recombinant plasmid. Colonies were grown on Luria-Bertani (LB)+Ampicillin (Amp) plates at 37 °C overnight. On the following day, monoclonal colonies were selected and inoculated in LB culture solution with Amp, and cultured in a constant temperature shaker set at 37 °C overnight. After colonies were selected, colony PCR and sequencing were performed to verify the construct.

#### Statement of ethics

This study was approved by the ethics committee of Zhejiang Provincial People’ Hospital. All procedures were performed according to the institutional guidelines for the care and use of laboratory animals. Mice were housed in a specific pathogen-free condition with adequate food and water. At the end of the study periods, a midline abdominal incision was performed after mice were anesthetized. Then, blood was collected from the abdominal aorta, and both kidneys were removed quickly. Finally, the mice were killed by cervical dislocation.

### Measurements

#### Western-blotting

The protein expression of ATF4, PERK, p-PERK, elF2α, and p-elF2α in different podocyte groups was detected by Western blotting. Briefly, cell lysates were collected in lysis buffer (Biyuntian Company, P0013B) containing Protein Stabilizing Cocktail (Thermo Fisher, 89806) and transferred onto PVDF membranes. After blocking with 5% fat-free dry milk in TBS-Tween (TBST), membranes were incubated with the following primary antibodies: PERK (Affinity, AF5304, 1:1000); p-PERK (Affinity, DF7576, 1:1000); elF2α (Proteintech, 12499-1-AP, 1:1000); p-elF2α (Affinity, AF3087, 1:1000); ATF4 (Affinity, DF6008, 1:1000); caspase-3 (CST, #9662, 1:1000); HO-1 (CST, #70081, 1:800); LC3 (CST, 4108S, 1:1000); and β-actin (Cell Signaling, #4970, 1:1000). After washing with TBST, membranes were incubated at 37 °C for one hour with an HRP-labeled secondary antibody (1:5000). After washed with TBST, blots were visualized using ECL reagent mixed with stable peroxidase solution at a ratio of 1:1, which was dripped onto PVDF membranes. A Tanon-5200 gel imaging system (Tanon) was used to visualize Western blots.

#### Detection of cellular apoptosis by flow cytometry

After digesting with 0.25% trypsin without EDTA, podocytes were collected and centrifuged at 1500 rpm for 5 min. After discarding the supernatant, e cells were resuspended twice in chilled PBS and centrifuged for 5 min at 1500 rpm. Cells were then mixed with 300 μL Binding Buffer suspension and 5 µL Annexin V-FITC in dark for 15 min. After that, 10 µL propidium iodide (PI) solution was added and suspensions were incubated in dark for another 10 min. Apoptosis was analyzed using a BD FACSCalibur flow cytometer (BD Biosciences).

#### Immunofluorescence staining

Podocytes were fixed with 4% paraformaldehyde for 15 min at room temperature and washed using PBS containing 0.1% Triton X-100 (Semberg, SBJ-1141). Cells were then blocked with 5% BSA (Hyclone, SH30574.03) in PBS for one hour. Primary antibody LC3 antibody (Mitrin Biology, 14600-1-AP, 1:50) or nephrin (Santa Cruz, sc-377246, 1:100) was added to the cells and incubated at 4 °C overnight. After extensive washing with PBS, cells were incubated with secondary antibody Alexa Fluor 594-conjugated Affinipure Goat Anti-Rabbit IgG (H + L) (Jackson, 111585003, 1:100) or Cy3 AffiniPure Goat Anti-Mice IgG (H + L) (Aimeijie, A22210, 1:100) at 37 °C in dark for 1 h. Cells were then washed, counterstained by DAPI for 5 min, and photomicrographed by fluorescence microscopy or laser scanning microscopy.

#### Determination of urinary protein, serum creatinine, and urea levels

A fully automatic biochemical analyzer was used to determine the urinary protein of mice at 13 weeks old (24 h before treatment) and at 25 weeks old (one day before the end of treatment). Blood was collected from the abdominal aorta and sit at room temperature for 2 h. After centrifuged at 1500 rpm for 10 min, the supernatant was collected. The urine creatinine test kit (Nanjing Jiancheng, C011-1) and urea nitrogen test kit (Nanjing Jiancheng, C013-2) were used to test the specific biofluid of interest, urine, pladma, and serum according to manufacturer’s instructions.

#### Periodic Acid-Schiff (PAS) stain

Formalin-fixed, paraffin-embedded tissue sections were dewaxed, rehydrated, and put into 1% aqueous solution of periodic acid for 10 min. After that, tissue sections were washed in tap water and then in distilled water. After stained with Schiff reagent (Leagene, DG0005) for 10 min, tissue sections were washed by flowing water for 10 min, and then stained with Harris hematoxylin for 10 min. After incubation with 1% hydrochloric acid-ethanol for 10 s and subsequent washes, the blue colorimetric reaction was performed by incubating samples with 1% ammonia for 10 s. PAS staining was analyzed under a microscope.

#### Terminal deoxynucleotidyl transferase-mediated dUTP nick end-labeling (TUNEL) staining

TUNEL has become a widely used staining method to help to detect apoptotic cells in tissue sections. Briefly, apoptotic cells were detected using a TUNEL Apoptosis Detection Kit (Biobox, BA27). Apoptotic cells were evaluated by microscope.

### Statistical analysis

All statistical analyses were performed using SPSS 23.0 software (Stanford University, Stanford, CA). Results are expressed as mean ± standard deviation (SD). Significance was determined by student’s t test or one-way ANOVA followed by the Duncan's multiple-range test for multiple comparisons. Non-normally distributed variables were compared using the Kruskal–Wallis test with Mann–Whitney U for post-hoc testing. All *p*-values are two-tailed, and *p* < 0.05 was considered to indicate statistical significance.

### Materials

#### Animal models

Eight-week-old C57BL/KsJ db/db mice exhibiting spontaneous DN and control C57BL/KsJ db/m mice (22 ± 2.5 g) were provided by Changzhou Canvas Laboratory Animal Co., Ltd. (SCX (Su) 2016-0010).

#### Drugs and reagents

The following reagents and drugs were used in this study: RPMI-1640 medium (Hyclone, SH30809.01B); collagenase IV (Gibco, 17104-019), PBS (Solebao, P1020); bovine serum albumin (BSA, Hyclone, SH30574.03); 1% Triton x-100 (Senbega, SBJ-1141); double antibody (Beijing Xia Si Biotechnology Co., Ltd., SV30010); 0.25% Trypsin (Gibco, 15050065); rapamycin (Targetmol, T1537); hemin (Sigma, 51280-1 G); γ-IFN (Sigma, I4777); FBS (Gibco, 16000-044).

## Results

### In vitro studies

#### Serum derived from diabetic nephropathy (DN) mice caused podocyte injury and increased ATF4 expression *in vitro*

**Figure 1.**
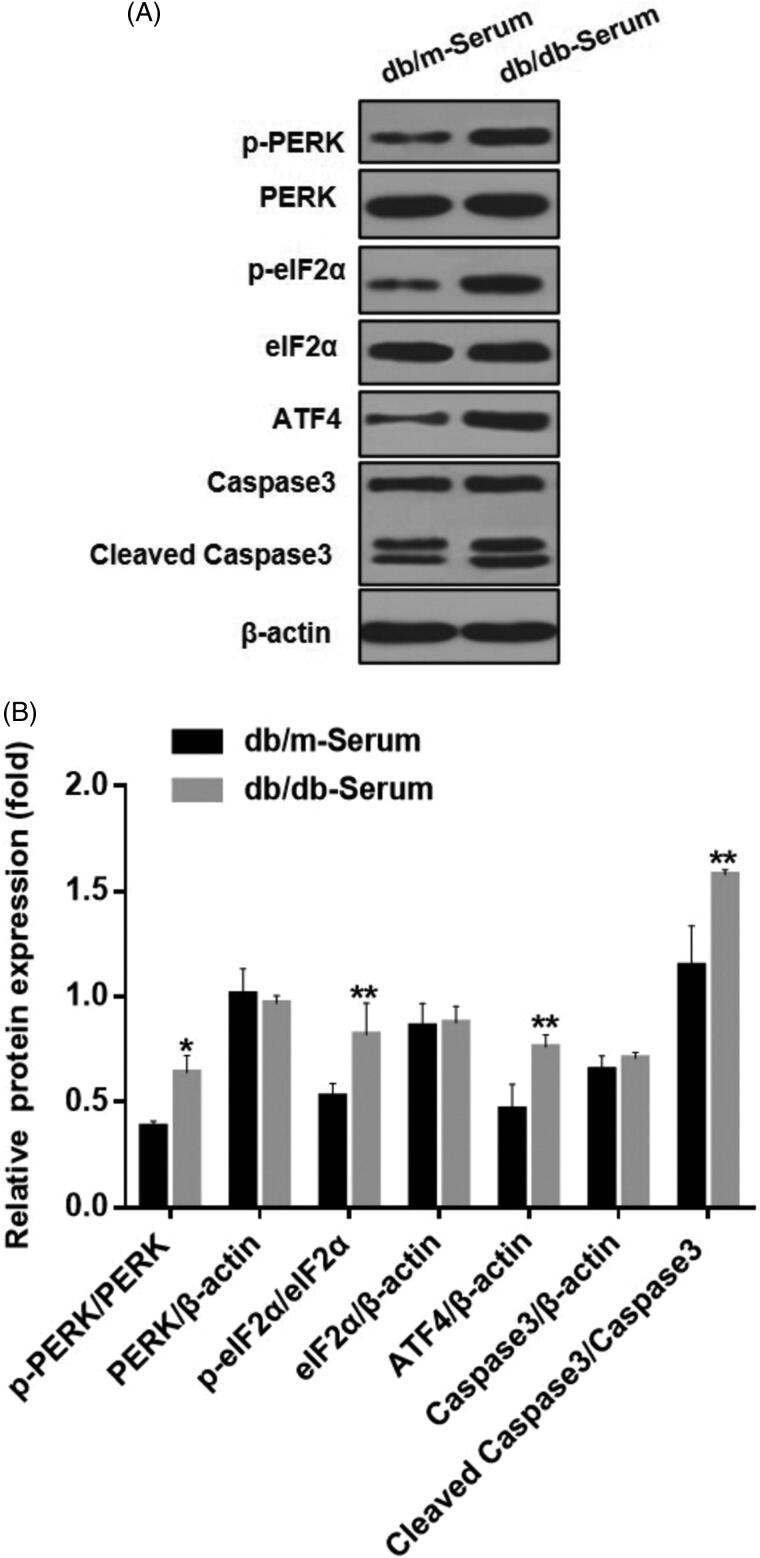
Serum from DN mice induced podocyte injury and endoplasmic reticulum stress. (A) MCP5 podocytes were treated either with 10% serum from control C57BL/KsJ dm/m mice or with 10% serum from C57BL/KsJ dm/dm DN mice for 24 h. Western blot image showing activation of PERK-eIF2α-ATF4 in ER stress signaling pathway and increased apoptosis-related molecule cleaved caspase-3 in podocytes treated with serum from DN mice as compared to control mice. (B) Densitometric quantification of protein expression from Figure 1(A). (**p < 0.01, *p < 0.05).

We examined the effect of serum derived from DN mice on podocytes ER stress *in vitro*. Compared to serum from healthy mice, exposure to serum from DN mice caused significant podocytes damage, increased expression of apoptosis marker cleaved caspase-3 protein, and up-regulation of p-PERK and p-eIF2α, indicating activated endoplasmic reticulum stress (Figure 1(A,B)). Additionally, expression of transcriptional activator of unfolded protein response (UPR) target genes, ATF4 was significantly increased. These results suggest that many features of ER stress including activation of PERK-eIF2α-ATF4 pathway, can be applied using this cellular model to study DN-induced podocyte injury (Figure 1).

#### ATF4 knockdown promotes podocytes apoptosis *in vitro*

**Figure 2.**
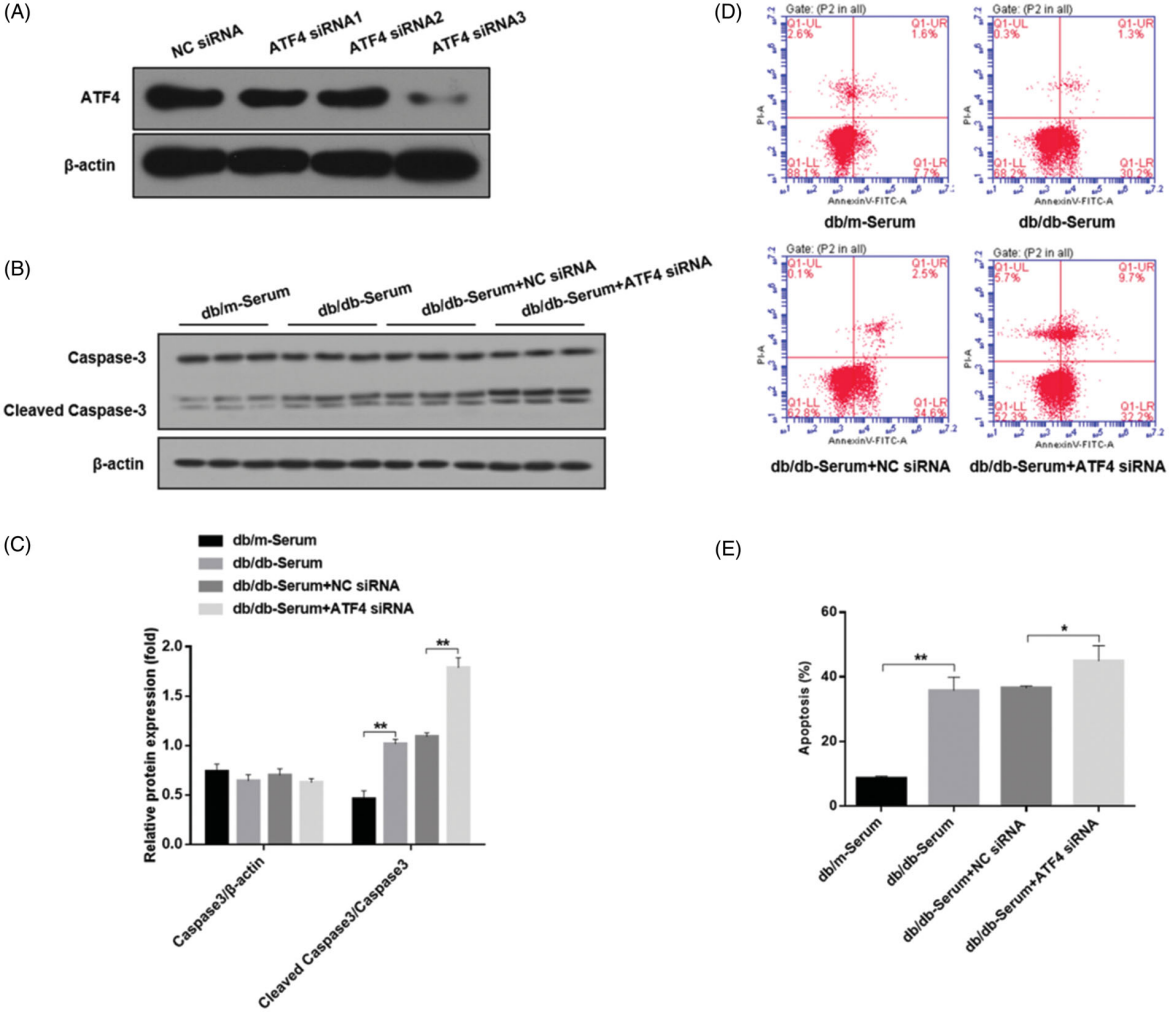
Effects of ATF4 knockdown on podocyte apoptosis when exposed to serum from DN mice in-vitro. (A) Comparison of knockdown efficiency of various ATF4 siRNA constructs and the most efficient ATF4 siRNA-3 was selected for the present study. (B) Western blot analysis showed that cleaved caspase-3 protein expression was markedly increased by ATF4 siRNA in MCP-5 cells. (C) Densitometric analysis of caspase-3 protein expression from Figure 2(B). (D) Podocyte apoptosis determined by flow cytometry showed significant increase in cell death by ATF4 siRNA treatment. (E) Quantification of apoptosis rates from Figure 2(D). siRNA-NC: Negative control siRNA. (**p < 0.01, *p < 0.05).

In order to determine the role of ATF4 on podocyte apoptosis, we employed gene knockdown strategy using four different ATF4-silencing small interfering RNAs, and selected the most efficient siRNA for our study (Figure 2(A)). Podocytes transfected with ATF4 siRNA displayed markedly higher cleaved caspase-3 and podocyte apoptosis when exposed to serum from DN mice as compared to negative control siRNA (Figure 2(B–E)), indicating important role of ATF4 in ameliorating podocytes apoptosis in DN.

#### ATF4 silencing decreased DN serum-induced autophagy in podocytes

**Figure 3.**
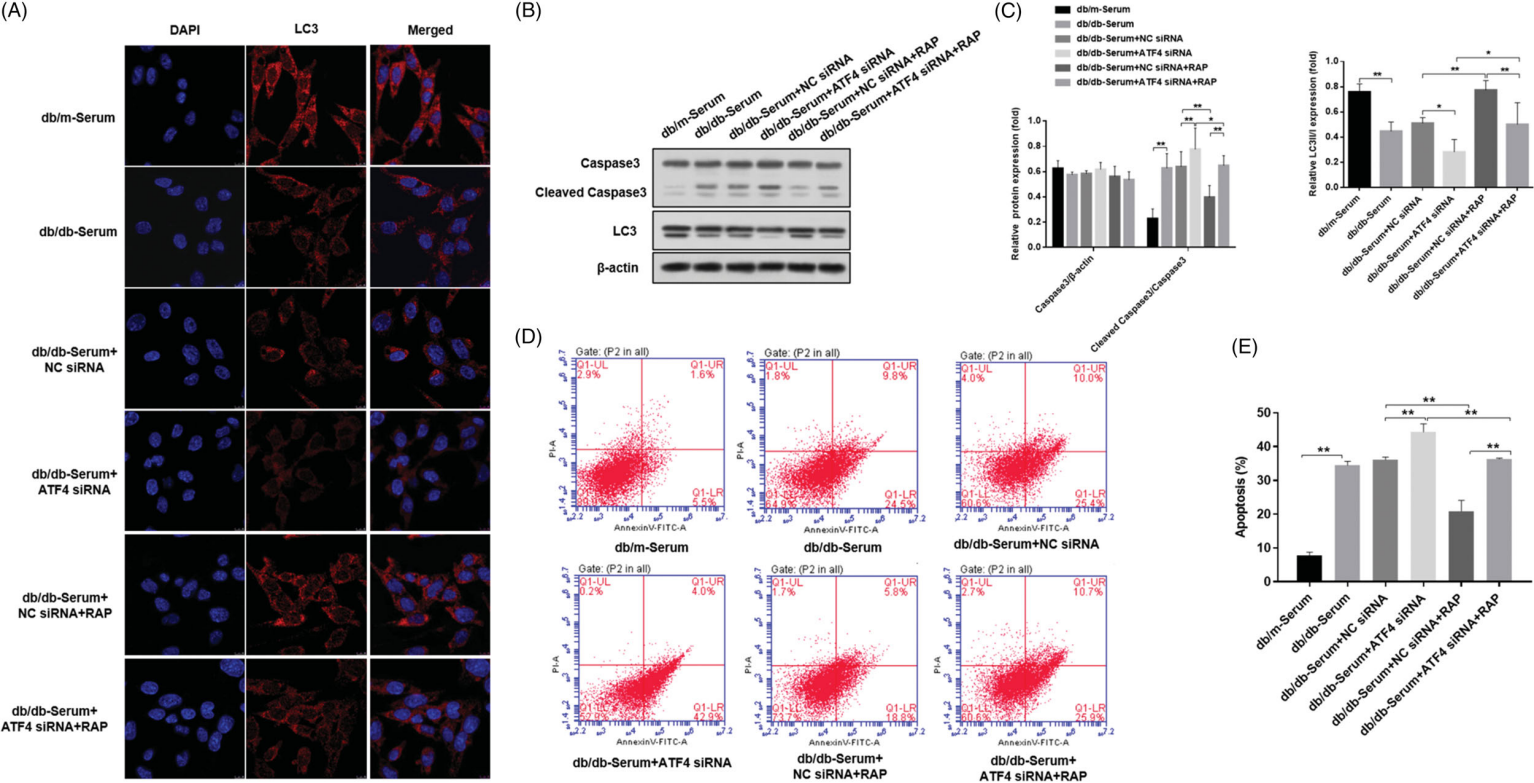
Effects of ATF4 knockdown and mTOR inhibitor rapamycin on autophagy and apoptosis in podocytes induced by serum from DN mice. (A) Immunofluorescence analysis of expression and distribution of autophagy markers protein LC3 showed significant downregulation by ATF4 siRNA in MCP-5 podocytes exposed to serum from DN mice, which was reversed by rapamycin treatment; scale indicates 10 μm. (B) Protein expression analysis supported our immunofluorescence data on LC3 downregulation by ATF4 siRNA and rescue by rapamycin. It further showed that rapamycin treatment significantly reduced cleaved caspase-3 expression. (C) Densitometric analysis of protein expression from Figure 3(B). (D) Densitometric analysis of protein expression. (E) Podocyte apoptosis was evaluated by flow cytometry. (F) Quantification of apoptosis rates. siRNA-NC: control siRNA transfected for 48 h; siRNA-ATF4: siRNA-ATF4 transfected for 48 h. RAP, Rapamycin treatment for 24 h. (**p < 0.01, *p < 0.05).

Autophagy plays an important role in the inhibition of podocyte injury induced by high glucose levels. While induction of autophagy can inhibit podocyte injury in the context of high glucose levels, role of eIF2α/ATF4 pathway of autophagy in podocytes of mice with diabetic nephropathy is not clear [19,26]. To investigate whether autophagy is the mechanism by which ATF4 impacts podocyte injury induced by DN mice serum, we examined the expression of autophagy markers LC3. Immunofluorescence analysis revealed that, treatment with DN mice serum significantly reduced LC3 punctate aggregates in podocytes, indicating a decrease in autophagy. ATF4 silencing led to further downregulation of LC3 punctate in podocytes, suggesting an even more pronounced reduction in autophagy. Our study further showed that treatment with mTOR inhibitor rapamycin reversed this effect, and LC3 punctate aggregates were markedly increased (Figure 3(A)). Western blot analysis showed that treatment with DN mice serum promoted cleavage of apoptosis-related caspase-3 protein and decreased the ratio of autophagy protein LC3II/I, and ATF4 silencing exacerbated these effects. Whereas Rapamycin treatment reduced the cleaved caspase-3 protein and increased the ratio of LC3II/I (Figure 3(B–D)). Apoptosis rate determined from flow cytometry analysis replicated and verified our findings of cleaved caspase-3 by western analysis (Figure 3(E,F)). Our study therefore suggests that knockdown of ATF4 promotes podocyte apoptosis by inhibiting autophagy and presumably, ATF4 positively regulates autophagy and inhibit podocytes injury.

#### ATF4 knockdown regulates podocyte autophagy and apoptosis via HO-1

**Figure 4.**
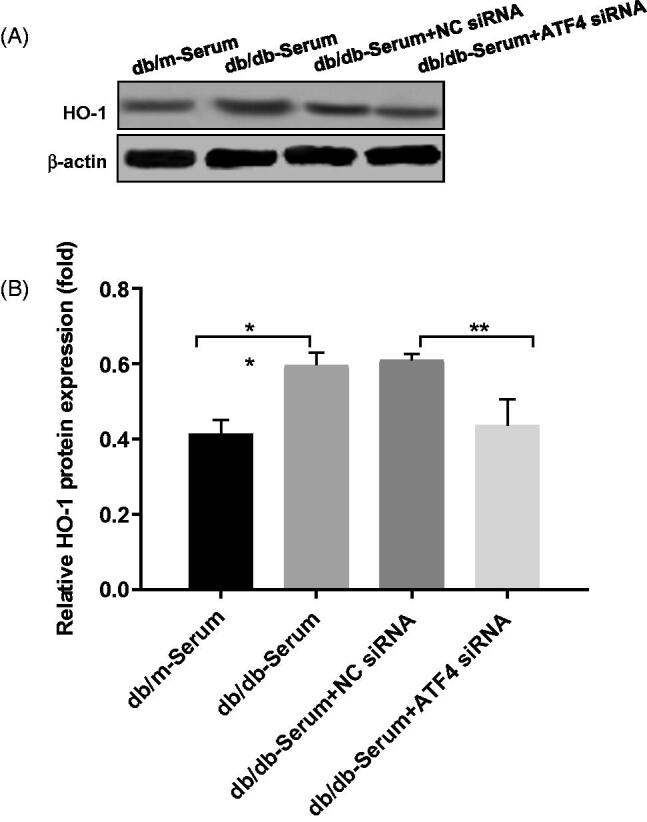
Effects of ATF4 knockdown on HO-1 expression on podocytes exposed to serum from DN mice. (A) Protein expression analysis revealed that HO-1 expression was increased by serum from DN mice, indicating increased ER stress, while ATF4 siRNA significantly reduced HO-1 expression in MCP-5 podocyte. (B) Densitometric analysis of protein expression from Figure 4(A) (**p < 0.01, *p < 0.05).

Studies showed that ATF4 regulates HO-1 expression [24], and HO-1 stimulates autophagy and inhibits podocyte apoptosis induced by high glucose [27], however role of HO-1/ATF4 axis in podocytes of diabetic nephropathy remains unknown. Our study showed that, exposure to DN mice serum caused up-regulation of HO-1, whereas HO-1 expression was decreased in podocytes after ATF4 silencing (Figure 4), suggesting a link between ATF4 and HO-1 expression in DN.

**Figure 5.**
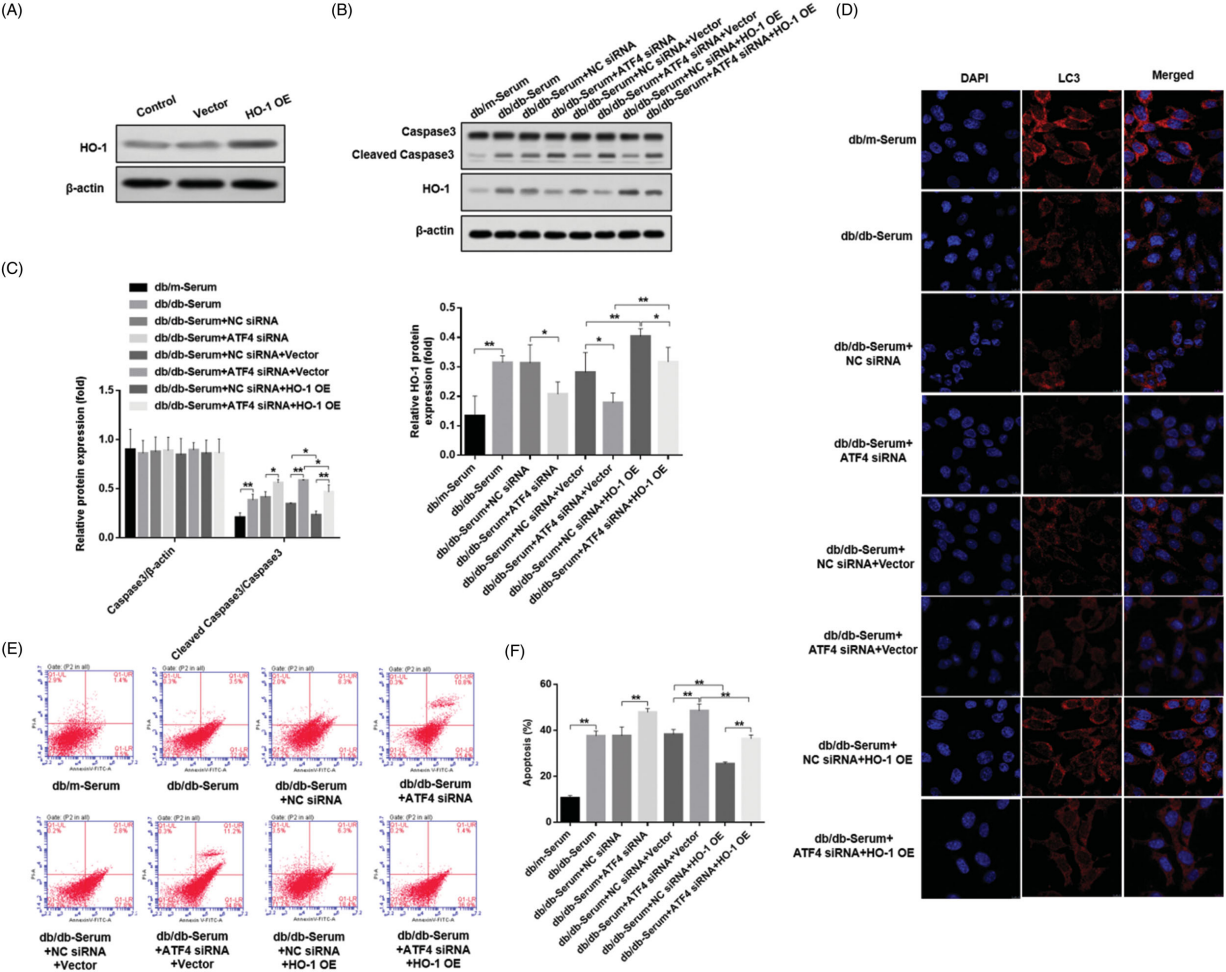
Effects of ATF4 knockdown and HO-1 overexpression on podocyte autophagy and apoptosis when subjected to serum from DN mice. (A) HO-1 overexpression was verified in MCP-5 podocytes. (B) Podocytes overexpressing HO-1 were subjected to serum from DN mice, showed significantly reduced cleaved caspase 3 expression, and co-treatment of HO-1 overexpression with ATF4 siRNA increased cleaved caspase-3 expression. (C) Densitometric analysis of protein expression from Figure 5(B). (D) Immunofluorescence analysis showed increase in autophagy LC3 protein expression by HO-1 overexpression, which was significantly reduced by ATF4 siRNA co-treatment; the scale indicates 10 μm. (E) Quantification of apoptosis rates by flow cytometry supported our western analysis of cleaved caspase-3. (F) Apoptosis was evaluated by flow cytometry. NC siRNA: control siRNA transfected for 48 h; ATF4 siRNA: siRNA-ATF4 transfected for 48 h. Vector: Empty plasmid transfected for 48 h. HO-1 OE: HO-1 overexpression plasmid transfected for 48 h. (**p < 0.01, *p < 0.05).

In order to verify whether ATF4 inhibits apoptosis through HO-1, we designed and synthesized a mouse-derived HO-1 sequence, constructed an overexpression vector, and verified the effect of HO-1 overexpression by western blot (Figure 5(A)). ATF4 siRNA and HO-1 overexpression vectors were transfected into podocytes. Western blot shows that HO-1 overexpression plasmid mitigated the down-regulation of HO-1 expression induced by ATF4 siRNA, and increased caspase-3 cleavage (Figure 5(B,C)). Immunofluorescence analysis showed that HO-1 overexpression is unchanged (Figure 5(D–F)). These data demonstrate that ATF4 inhibits podocyte apoptosis in DN by HO-1 regulation of autophagy.

### In vivo studies

#### Therapeutic effects of HO-1 agonist in mice with DN

**Figure 6.**
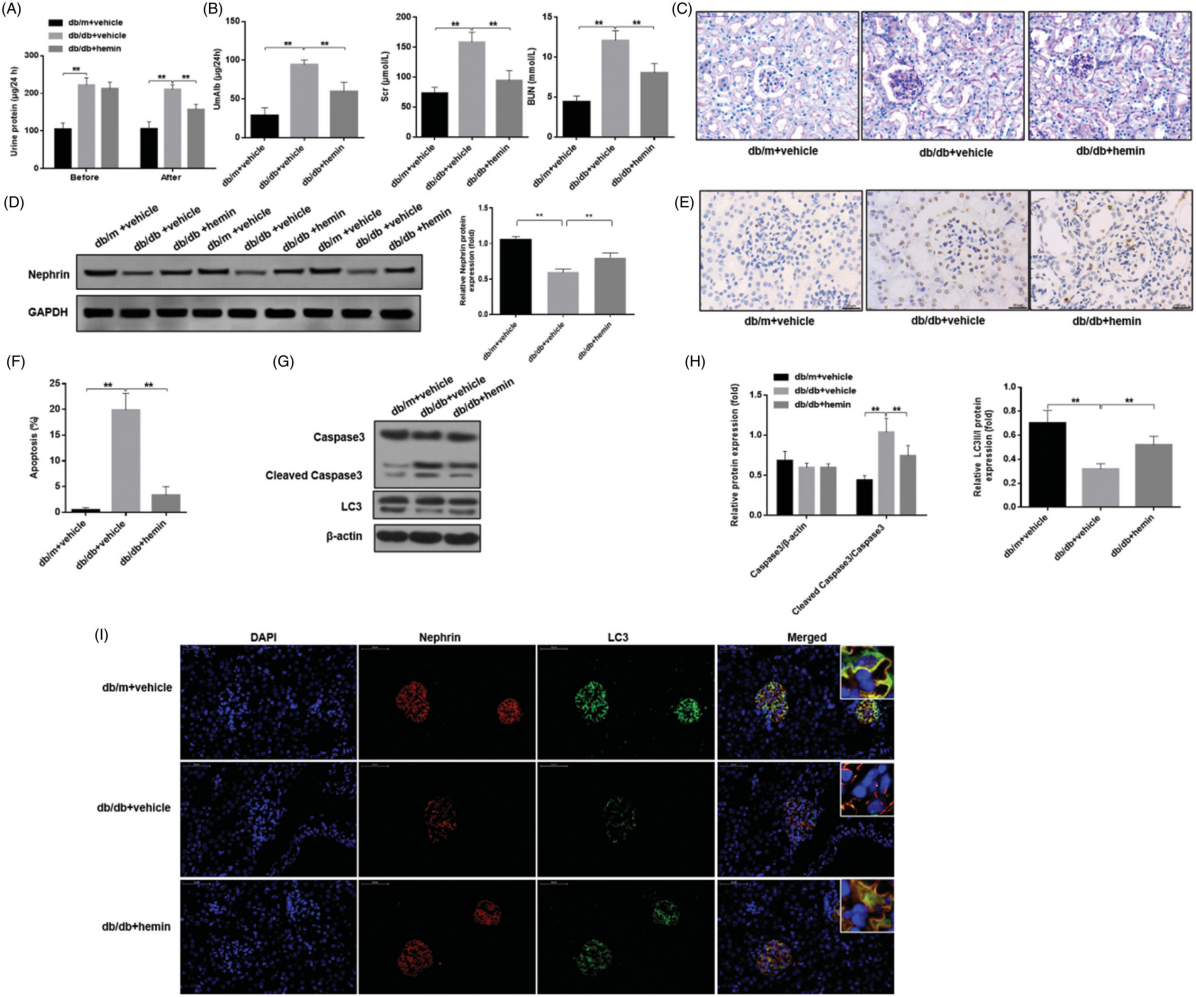
Effects of HO-1 agonist hemin treatment on clinical indications of DN-induced autophagy and apoptosis in podocytes. (A) Evaluation of urinary total protein and urinary albumin showed increased content in DN mice compared to healthy controls, while hemin treatment significantly reduced proteinuria and albuminuria in DN mice. (B) Serum creatinine and urea levels were also significantly reduced by hemin treated DN mice. (C) PAS staining showed glomerular damage in DN mice and it was rescued by hemin treatment. (D) Protein expression analysis revealed that expression of critical protein in renal filtration barrier nephrin was downregulated in DN mice, and hemin treatment significantly increased the neprhin expression and densitometric analysis of protein expression. (E) TUNEL staining showed that hemin treatment significantly reduced apoptotic cells in DN mice. (F) Quantification of apoptotic cells. (G) Protein expression by Western blot. (H) Densitometric analysis of protein expression. (I)Expression and distribution of nephrin and LC3 protein were evaluated by immunofluorescence; the scale indicates 50 μm. (**p < 0.01, *p < 0.05).

We determined in our *in vitro* experiments that ATF4 inhibited podocyte apoptosis induced by serum from DN mice through autophagy induced by HO-1. Based on our initial *in-vitro* data showing reduced ATF4 expression promoted apoptosis and reduced HO-1 expression in podocytes exposed to serum from DN mice, we performed *in-vivo* study using DN mouse model to study the therapeutic effects of HO-1 agonist hemin to determine its role in podocyte apoptosis and autophagy. Previous study has shown that the expression of ATF4 were highly elevated in the db/db mice compared with that in the db/m mice [1]. In our research, we detected the expression of HO-1 and find that the expression of HO-1 was increased in DN mice, whereas hemin treatment significantly increased it. (Figure S1) Then, we evaluated urinary total protein and urinary albumin content in hemin treatment. Before the onset of the treatment, and as expected DN mice exhibited proteinuria compared to the healthy control mice. No difference in urinary total protein was found between the agonist-treated and untreated DN mice groups. At the end of the hemin treatment, we found that hemin exposure significantly decreased proteinuria as well as albuminuria compared to untreated DN mice (Figure 6(A)). Additionally, analysis of blood chemistry revealed that serum creatinine and serum urea levels were increased in DN, compared to healthy control mice, and hemin treatment significantly reduced their levels (Figure 6(B)). These data suggest beneficial therapeutic effects of HO-1 agonist hemin in alleviating renal damage in DN.

We next evaluated renal tissues for glomerular damage in hemin treatment. In healthy mice, PAS staining showed that the glomerular structure of control mice was clear, and extracellular matrix and mesangial cells were normal in morphology. In DN mice, modest increase in glomerular volume, thickening of mesangial matrix, and mesangial cells proliferation were noticed. After hemin treatment, glomerular volume, mesangial matrix thickening and mesangial cell proliferation were reduced (Figure 6(C)). We also found that glomerular injury as assessed by measuring the expression of nephrin, an important renal filtration barrier protein in DN mice was downregulated significantly, and hemin treatment significantly increased nephrin expression, attenuating glomerular injury (Figure 6(D)). TUNEL assay showed that apoptosis of glomerular cells in DN mice was increased significantly, and hemin treatment significantly reduced apoptosis of glomerular cells (Figure 6(E,F)).

We further evaluated glomerular podocyte injury. Based on our data, we hypothesized that HO-1 may inhibit podocyte apoptosis induced by DN mice serum through promoting autophagy. In order to elucidate the mechanism of HO-1 action, we measured autophagy and apoptosis markers in hemin treated renal tissues used autophagy fluorescence detection and western blot. Glomerular LC3 punctate aggregates and the LC3II/I ratio in DN mice were significantly decreased compared to healthy control mice, and hemin treatment significantly increased LC3 punctate and LC3II/I ratio and inhibited apoptosis (Figure 6(G–I)). These results were consistent with the *in vitro* experiments and showed that hemin likely inhibits apoptosis of podocytes by inducing podocyte autophagy to alleviate renal lesions and improve renal function in this DN model.

## Discussion

In this study, we used both in-vitro cellular model and *in vivo* mouse model to investigate the role of ATF4 and HO-1 in podocyte injury caused by DN. We demonstrated that exposure to serum from DN mice promoted podocyte injury, activated ER stress, and increased ATF4 expression. We demonstrated that siRNA-mediated knockdown of ATF4 exacerbated DN-induced podocyte apoptosis and markedly reduced autophagy response, which were partially rescued by the mTOR inhibitor rapamycin. We also showed that ATF4 knockdown significantly decreased the HO-1 expression in the podocytes. Using diabetic mouse model, we demonstrated that treatment with HO-1 agonist, hemin, markedly attenuated the DN-induced renal injury as evidenced by reduced albuminuria, ameliorated glomerulosclerosis and increased nephrin expression. The protective effect of hemin was associated with reduced renal apoptosis and enhanced autophagy response. This study highlights the protective role of ATF4 and HO-1 against DN-induced podocyte injury.

Activation of ER stress is increasingly investigated in various physiological and pathophysiological conditions and experimental evidence indicates that ER stress is involved in the glomerular damage associated with diabetic nephropathy [1]. ATF4 is a major regulator of ER stress and is a part of PERK-eIF2α-ATF4 unfolded protein response (UPR) in DN. Emerging evidence indicate that ATF4 plays critical role in regulating ER stress, autophagy and apoptosis. In our study, serum of DN mice caused significant increase in ATF4 expression, paralleled by marked induction of UPR signaling pathways and ER-induced apoptotic markers. This data indicates that serum of DN mice is sufficient to cause activation of many ER features in our cellular model. Interestingly, knockdown of ATF4 dramatically triggered podocyte apoptosis and abolished autophagy response as evidenced by increased cleaved caspase-3 expression and reduction in autophagy markers LC-3, respectively. The data are in agreement with previous report showing activation of podocyte ER stress caused by high-glucose.

Guan Bo-Jhih *et al.* showed that eIF2α/ATF4 signaling promotes mTORC1 activity, and demonstrated that mTORC1 is a downstream target of eIF2α/ATF4 signaling [28]. Additionally, inhibition of mTOR signaling by rapamycin is shown to prevent apoptosis and thus may regulate autophagy [4]. Supporting previous findings, our data showed that rapamycin rescued DN serum-induced apoptosis and promoted autophagy response in both non-transfected and ATF4 silenced podocytes, however recuse response was lower in ATF4 knockdown by rapamycin, consistent with the role of protective role of ATF4 in this study. Our results suggest that ATF4 is a critical player in ER stress in DN-induced podocyte injury and indicates role of mTORC1 in ATF4 mediated protective autophagy response in podocytes.

Heme oxygenase-1 (HO-1) plays pivotal role in regulating autophagy and its expression increases under stress conditions. HO-1 is shown to ameliorate oxidative stress [29] and improves renal function in DN [30]. In the present study, we investigated the role of HO-1 and ATF4 in DN-induced ER stress. We found that HO-1 expression was significantly increased in podocytes exposed to serum from DN mice suggesting activation of self-protection mechanism and ATF4 knockdown significantly reduced HO-1 expression, indicating HO-1 expression may be regulated by ATF4 in podocytes in diabetic nephropathy. This data is in agreement with previous report showing association between HO-1 expression and transcription factor ATF4 in tumor [2]. Additionally, we demonstrated that HO-1 overexpression attenuated DN-induced podocyte apoptosis and triggered autophagy response. Furthermore, ATF4 knockdown prevented the beneficial effects of HO-1 overexpression in podocytes. This data suggests that effects of ATF4 in podocyte injury are mediated at least in part *via* HO-1-regulated autophagy.

Using *in vivo* mouse model of diabetic nephropathy, we evaluated the effect of hemin, an inducer of HO-1 on renal apoptosis and autophagy. In our study, C57BL/KsJ db/db mice developed progressive proteinuria and elevated serum creatinine and BUN, as well glomerulosclerosis similar to human DN, indicating the renal dysfunction and the development of DN in our animal model. Interestingly, hemin treatment significantly attenuated albuminuria, reduced serum creatinine, BUN and ameliorated glomerular histopathology, suggesting hemin effectively prevented the development of DN. Furthermore, our results revealed that hemin alleviated number of renal apoptotic cells and markedly enhanced the expression of nephrin and autophagy marker LC-3. This data suggest that HO-1 protects DN-induced ER stress in podocyte injury by enhancing autophagy response.

## Conclusions

This study provides evidence that ATF4 protects against DN-induced podocyte injury by mechanisms involving attenuating PERK-eIF2α-ATF4 pathway in ER stress mediated by mTORC1 inhibition and autophagy enhancement promoted by LC-3 upregulation. Additionally, HO-1 exerts beneficial effects in ameliorating renal apoptosis and enhanced autophagy in mouse model of DN, which subsequently improved albuminuria and glomerulosclerosis and prevents the progression of DN. This study highlights the important role of ATF4 and HO-1 in regulating renal apoptosis and autophagy, and HO-1 inducers may represent promising therapeutic strategy for preventing DN development.

## Supplementary Material

Supplemental Material
