# Supplementary figures and images for: ATF4-dependent heme-oxygenase-1 attenuates diabetic nephropathy by inducing autophagy and inhibiting apoptosis in podocyte
Source: Ren Fail. 2021 Jun 22;43(1):968–79. doi: 10.1080/0886022X.2021.1936040 (PMC8231401; doi:10.1080/0886022X.2021.1936040)

Figure S1

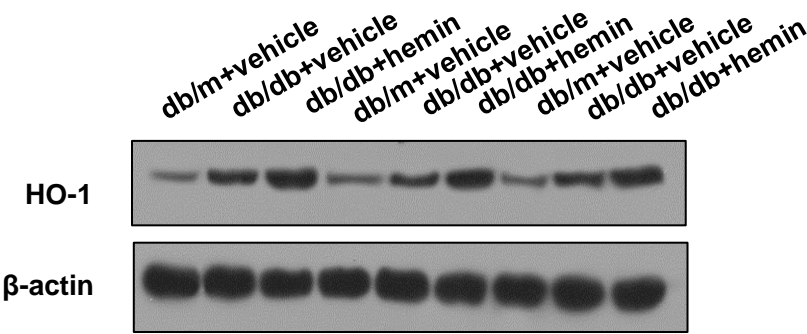

Supplement: Supplemental Material [file IRNF_A_1936040_SM3503.pdf]
